# The Role of Coronary Collaterals in Chronic Total Occlusions

**DOI:** 10.2174/1573403X10666140311123814

**Published:** 2014-02

**Authors:** Gerald S. Werner

**Affiliations:** Medizinische Klinik I (Cardiology & Intensive Care), Klinikum Darmstadt GmbH, Grafenstrasse 9, D-64283 Darmstadt, Germany

**Keywords:** Coronary artery disease, Stable angina pectoris, Chronic coronary occlusion, Collateral circulation.

## Abstract

A chronic total occlusion (CTO) describes a completely occluded coronary artery. This type of lesion is found in about 18% of all significant lesions in patients with coronary artery disease. A system of collateral connections are observed in almost all of these lesions, which have the capacity to prevent myocardial necrosis and may even uphold metabolic supply to the territory distal to an occlusion to maintain full contractile capacity. During exercise these collaterals are limited in their functional reserve, and more than 90% of patients with a well collateralized occlusion will experience ischemia. in the absence of ideal animal models that mimic the human collateral circulation, we need to rely on studies in man. The knowledge of collateral physiology in man has increased considerably over the past two decades with the advent of intracoronary sensors of coronary pressure and flow velocity. A number of basic physiologic questions have been answered by these studies. The blood supply through coronary arteries depends on a complex array of in general serial resistance systems, with an additional array of multiple parallel resistances on the collateral level.

There seems to be a great interindividual variability in the ability to recruit preformed collateral connections in the case of an epicardial occlusion. Collateral function can develop to a similar functional level in patients post myocardial infarction with large akinetic territories as it does in patients with normal preserved regional function. The presence of viability is thus not a prerequisite for collateral development. The question of practical relevance in the era of interventional therapy of chronic occlusions is also, whether a patient with coronary artery disease will remain protected by collaterals after removing the obstruction in the collateralized artery, or whether collaterals regress and lose their functional capacity. Both developments are observed again mainly depending of individual predisposition.

## INTRODUCTION

A chronic total occlusion (CTO) describes a completely occluded coronary artery. In order to find a common ground for future discussion of technique and patient outcome, a consensus was recently published by a group of European experts suggesting a firm definition of CTOs as those occluded arteries with a documented duration of occlusion of at least 3 months with absolutely no flow through the lesion (TIMI 0 flow) [1]. Occlusions of 1-3 months duration can be addressed as recent occlusions, and within 4 weeks after an acute myocardial infarction, as subacute occlusions. This definition and categorization is of particularly relevance when discussing the collateral function in this subset of coronary lesions.

The data on the prevalence of CTOs in patients with coronary artery disease vary between 20-30% [2-4] In a recent registry of more than 16000 diagnostic angiographies performed in 3 institutions in Canada, the prevalence was found to be 18.4% of all significant lesions in patients with coronary artery disease [5]. 

In general, patients with a CTO present with stable angina pectoris except if other coronary lesions progress and lead to unstable angina. They pose a high risk for all situations where the collateral supplying artery is involved in an acute myocardial infarction [6, 7], or in elective PCI for example of the left main stem in the presence of a CTO of the right coronary artery [8], as the territory at risk is increased.

## COLLATERAL CIRCULATION IN CTOS

Collaterals are interarterial connections that provide blood flow to a vascular territory whose original supply vessel is obstructed. Thus, the integrity of the organ supplied by the obstructed vessel may be preserved partly or completely. In the coronary vascular system such connections are familiar to every investigator who performs angiographic imaging of patients with coronary artery disease. They develop through arteriogenesis, that is, through the recruitment of preformed and pre-existing interarterial connections mainly driven by shear forces along the pressure gradient that develops when the native vessel is occluded [9]. Some of these connections may be preformed to such an extent that they are immediately recruitable during vessel occlusion, as shown during balloon occlusion in non-diseased coronary arteries [10]. The functional assessment of collaterals as mentioned below has revealed that in patients without well-developed pre-existing collateral connections, collaterals require between 2-12 weeks to fully develop their functional capacity [11].

The size of the interarterial connections varies over a wide range, between 40 and 200µm, but some inborn connections may have the size of coronary side branches well above 1 mm in diameter. However, the size of the majority of these connections is below the spatial resolution even of analogue angiographic imaging chains. Even with today’s digital storage media and a resolution of >0.2mm, quantitative coronary angiography of collaterals, which would be ideal, is limited. 

The classical and widely used angiographic grading system described by Rentrop, *et al*. does not actually rate the collaterals themselves, but their effect in filling the occluded arterial segment [12]. It distinguishes four degrees of collateral recipient artery filling by radiographic contrast medium: *grade 0*=no collaterals; *grade 1*=side branch filling of the recipient artery without filling of the main epicardial artery; *grade 2*=partial filling of the main epicardial recipient artery; *grade 3*=complete filling of the main epicardial recipient artery (Table).

A semi-quantitative assessment of collaterals in patients with CTOs has been introduced and validated with reference to physiologic measures of collateral function using a new grading method of collateral connections (CC grade 0=no continuous connection between collateral supplying and receiving vessel; CC1=thread-like continuous connection; CC2=side-branch like connection) [11]. This grading is also the basis of the assessment of potential collateral access in planning a coronary intervention for a CTO using the retrograde recanalization approach (Table).

## THE PHYSIOLOGIC ASSESSMENT OF COLLATERAL CIRCULATION IN CTOs

**Fig. (1):**
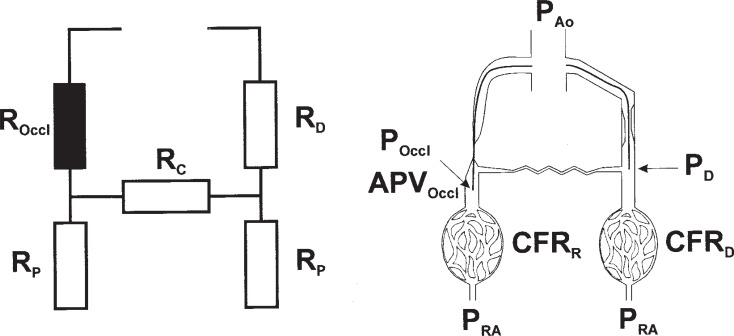
Schematic presentation of the electric analog model of coronary and collateral circulation (left), and of the experimental setup (right). Mean aortic pressure (PAo) is recorded via the guiding catheter. Pressure at the takeoff of the collateral in the donor artery (PD) is recorded before recanalization, as well as the coronary flow reserve (CFR)D in the donor artery. Collateral blood flow velocity (APVOccl) and pressure (POccl) are recorded distal to the occlusion before balloon dilatation, and CFRR in the recanalized artery at the end of the procedure. The resistance of the occlusion (ROccl) is infinitesimal, and resistance indexes are calculated to describe the donor (RD) and collateral resistance (RC), and the microvascular resistance distal to the occlusion (RP). PRA = mean right atrial pressure. Reprint from 27 with permission.

The blood supply through coronary arteries depends on a complex array of in general serial resistance systems, with an additional array of multiple parallel resistances on the collateral level. The main components are the resistance of the epicardial artery, the microvascular resistance of the arteriolar bed, and the collateral supply of blood. With a complete and chronic occlusion of the epicardial artery, the resistance of this segment is infinite, therefore the blood supply rests solely on the collateral connections and the microvascular resistance (Fig. **1**). However, the collateral supply is provided through a variety of anatomic connections, as a network of parallel resistances with varying dimensions. A simple assessment of the intracoronary pressure distal to an obstruction or occlusion does not give a correct picture of the collateral hemodynamics [13, 14]. The microvascular resistance is an integral part of this system and can only be assessed with the additional information of coronary flow.

While the physiologic aspects of collateral circulation and the methodological approach are discussed elsewhere in this collection of reviews it needs to be emphasized that the assessment of collateral function in CTOs has a different quality than in non-occluded lesions. The main difference as compared to the published studies in non-occluded arteries is the presence of an equilibrium of collateral supply at the time of assessment in CTOs, whereas there are many factors influencing the assessment of immediately recruited collaterals during an acute occlusion of a previously non-occluded artery. This equilibrium in a CTO can only be assessed if at the time of advancing microsensors distal to the occlusion the antegrade flow has not yet been re-established. This can only be ensured by passing occlusive microcatheters over a typical recanalization guidewire and then advanced further through the occluded segment to enable an exchange for the pressure and flow wires.

The first use of the Doppler wire to assess the effect of a coronary occlusion on increased flow in the collateral supplying artery was elegantly demonstrated by Jan Piek in Morton Kern’s lab [15]. The direct evaluation of collateral flow velocity was made possible through the advancement of the Doppler wire beyond an occluding balloon and thus showing the phasic flow profile of the collateral supply. In many cases this was of a distinctly higher magnitude during systole; which is distinctly different from the flow profile of the native coronary artery with a more pronounced diastolic contribution [16-18]. 

In 1993, Nico Pijls and co-workers provided the experimental basis for the determination of maximum coronary, myocardial and collateral blood flow by coronary pressure measurements [19]. It was assumed that minimal microvascular resistance during pharmacologically-induced hyperaemia is constant as well as independent of the presence or absence of an epicardial stenosis. Both assumptions represent important limitations [20]. Following on from these studies the fractional collateral flow reserve can be deduced in a completely occluded artery, either naturally in the case of a chronic total coronary occlusion or iatrogenically by a balloon [21]. The great advantage of the fractional flow reserve concept for the practical use in interventional cardiology is the reliable evaluation of the physiologic measure of perfusion from a singular pressure recording distal to a coronary lesion. The prerequisite, however, for this approach to work is the induction of maximum hyperemia.

## THE INFLUENCE OF MAXIMUM HYPEREMIA ON COLLATERAL FLOW: CORONARY STEAL

Ever since the early studies, maximum hyperemia has been believed to be induced by the very fact of balloon occlusion alone inducing ischemia distal to the occlusion, and thus obviating the need for a specific hyperaemic stimulus such as systemic adenosine. Furthermore, there has been some debate about the interaction of epicardial lesion severity and microvascular resistance [22, 23]. In non-occlusive coronary lesions this assumption may be true, at least in patients with moderate or poor collateral supply, but if we study patients where we do not observe symptoms of ischemia due to a good collateral supply, maximum hyperemia may not be inducible and therefore these pressure recordings cannot be used reliably on their own as a simple measure of collateral perfusion [24]. 

Another fact that makes it obvious that pressure recordings alone yield an incomplete picture of the hemodynamics in the collateralized territory distal to an obstruction or occlusion is the serial arrangements of the 3 major conductance pathways integrated in this circulation model. Aside from the conductance through the collateral proper, which is determined by the length and diameter of these collaterals and which may often show a tortuous vessel course, the conductance in the segment of the collateral donor artery has to be taken into account, where diffuse atherosclerosis may impede flow to the collaterals, and last but not least the conductance of the arteriolar ramifications of the microcirculation of the myocardium distal to the occlusion [25-27]. 

One misconception is, that collateral perfusion may occur through the microcirculation, however, the inflow from collaterals always circumvents the arteriolar bed and enters directly into the epicardial level of the occluded artery. This can be demonstrated by the simple measurement of oxygen content of the blood arriving in the occluded territory. In our lab we can measure invariably an oxygen saturation within the normal range of 95% and above (personal unpublished data). A more important problem in understanding and interpreting the physiologic measures made distal to an occluded artery is the assumption that the conductance of the microcirculation is constant and during occlusion may be maximally increased (the hyperemic response).

The selective assessment of the response of both pressure recordings and flow velocity recordings distal to an occlusion as well as the pressure drop along the collateral donor segment before and during pharmacologically-induced maximal hyperemia demonstrated the diversity and individuality of the changes in collateral and microcirulatory conductance [27]. We have observed not only patients who have a retained reserve to increase flow to the occluded territory, but also about 40% of patients, who show a paradoxic decrease in flow: the classic phenomenon of “coronary steal”. The major difference between the two groups is the responsiveness of the microcirculation: that is, either a reserve in microcirculation for further increase in conductance, or in case of patients with steal, already a maximally increased conductance at baseline. There are most probably many factors involved including the general property of the microcirculation in the individual patient as well as in the donor artery territory, but among other factors we have observed more pronounced regional dysfunction in patients with the 'steal' phenomenon [27].

A close correlation between pressure and flow measures of collateral function were only found in patients with predominantly recruitable collaterals, not chronically occluded arteries, such as with the acute occlusion model approach during coronary balloon occlusion [28]. These patients had no previous myocardial infarction in the territory studied, and showed normal left ventricular function. As these prerequisites are found not in all patients, and the more relevant questions need to be answered in more complex patient settings, a combined use of pressure and flow velocity assessment is mandatory [24]. Unfortunately, partly due to the lack of further development of the Doppler wire technique, this method of a combined assessment of both pressure and Doppler assessment is rarely applied nowadays, and no publications can be found in the past 5 years. The technical refinement of a combined Doppler and pressure wire, which had been developed by Volcano Corp. (San Diego, USA), would be a major step to bring back this combined assessment of collateral function into the catheterization lab.

One needs to scrutinize these methodological subtleties when evaluating the experimental data published on the human collateral circulation. Most of the publications do not follow these recommendations, and any further efforts to find correlations with genetic markers for collateral development, or try to modify collateral function through medical interventions cannot yield meaningful results with an imperfect assessment of the major determinant of collateral conductance.

## FUNCTIONAL CAPACITY OF COLLATERALS IN CTOs: RELATION TO VIABILITY AND ISCHEMIA

Collateral function can develop to a similar functional level in patients post myocardial infarction with large akinetic territories as it does in patients with normal preserved regional function. The presence of viability is thus not a prerequisite for collateral development. This is in accordance with experimental studies on arteriogenesis, namely that the pressure drop along preformed interarterial connections is the driving force that recruits these connections in the case of an occlusion of the native artery [29].

**Fig. (2):**
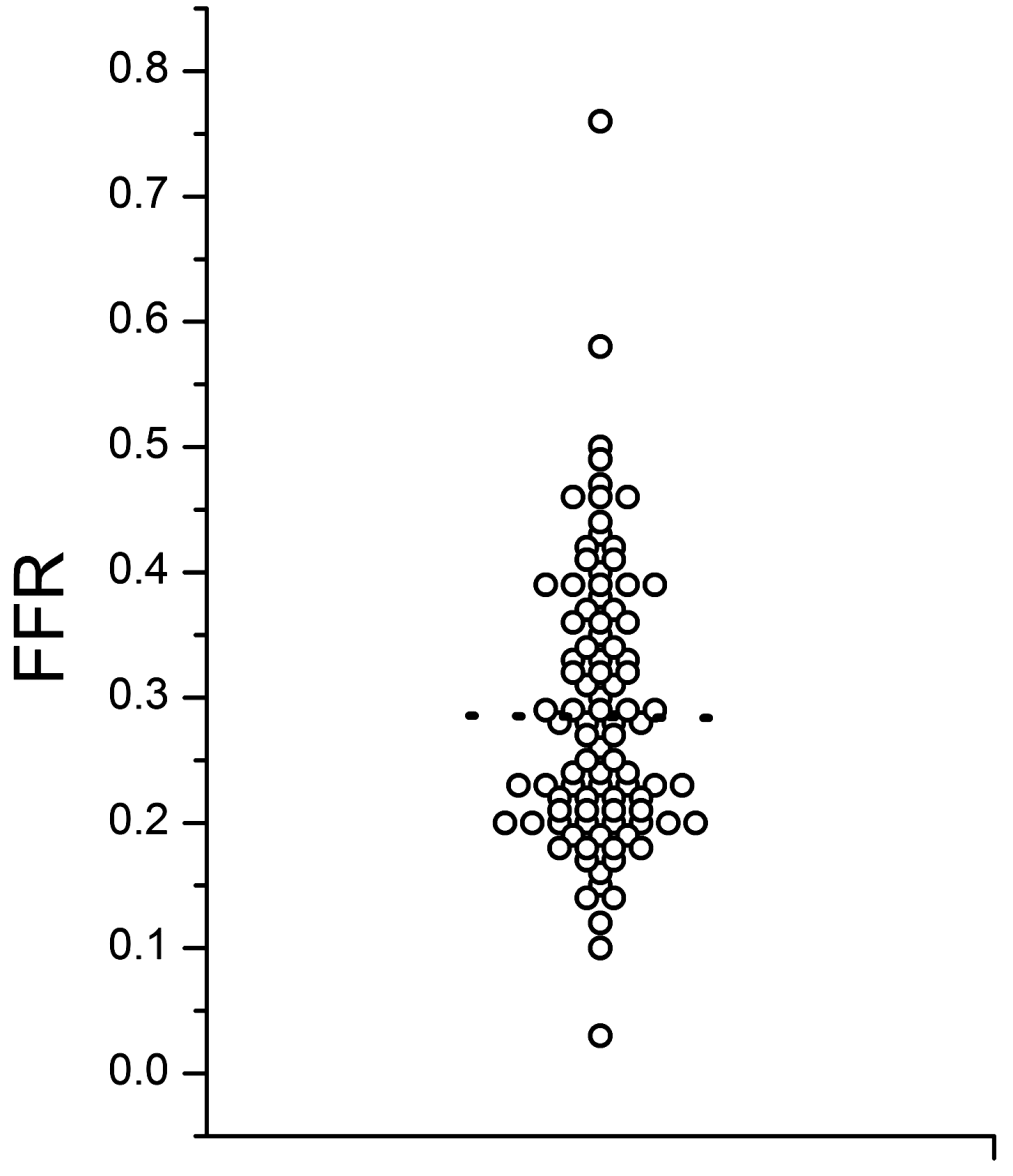
The distribution of fractional flow reserve (FFR) measured distal to a CTO during systemic adenosine infusion, based on data published in 30. The dotted line indicates the mean value of all 94 measurements of 0.29.

We know that collaterals have the capacity to prevent myocardial necrosis and may even uphold metabolic supply to the territory distal to an occlusion to maintain full contractile capacity. But direct assessment of collateral function shows that the functional competence of collaterals in chronic total coronary occlusions is limited even in patients without a prior Q-wave MI. During a standard stress protocol with systemic infusion of adenosine the coronary flow velocity and pressure changes distal to an occlusion are well below the cut-off values for assessing the functional reserve in non-occlusive coronary obstructions (i.e., FFR above 0.75) (Fig. **2**). Therefore, even well developed collaterals would not prevent ischemia during exercise [26, 27, 30].

An additional aspect of the functional capacity of collateral supply is the fact that the extent of atherosclerotic disease of the collateral donor artery. Among patients with coronary steal, the FFR measured in the collateral donor artery proximal to the collateral take-off is significantly lower than in patients without any coronary steal [27]. On the other hand, the FFR measured at the site of the collateral donor artery is also determined by the amount of myocardium supplied by this artery [19]. In the case of a large and viable collateral supplied area a lesion within the donor artery may in fact yield a lower value of FFR as compared to the same lesion without a collateral dependent additional myocardial territory. This was elegantly proven by observations where FFR was measured before and after recanalizing the collateral dependent territory [31].

## COLLATERAL FUNCTION DURING AND AFTER REVASCULARIZATION

Studies in dogs have shown a regression of collaterals after restored perfusion, and a capacity to recover during a prolonged reocclusion [32, 33]. The applicability of these data to human pathophysiology was not shown until the advent of direct measurement of collateral function in man with miniaturized sensors of coronary flow and pressure as described above. Possible clinical determinants of collateral regression could be diabetes mellitus, the regional LV function and myocardial viability, or angiographic factors such as collateral anatomy and size, and of course the incidence of restenosis or reocclusion. 

The question arising from clinical observations is whether a patient with coronary artery disease will remain protected by collaterals after removing the obstruction in the collateralized artery, or whether collaterals regress and lose their functional capacity. The incidence of MI in case of reocclusion after a successful recanalization in recent prospective studies is evidence for a collateral regression [34, 35]. However, the rate of MI was smaller than the incidence of reocclusion. This could be explained by a potential for collateral recruitment or persistent collateral channels in some patients [36, 37]. 

One of the main reasons for sometimes confusing and divergent study results in man relating to the regression of collateral function is caused by the heterogeneity of different patient settings. It is most notable that this confusion leads to contradictory main outcome messages even today, when one group reports on preserved recruitability of collateral function after revascularization, whereas another group reports on collateral functional loss [38, 39]. Both observations bear some truth in a specific and distinctly different setting, which had been shown already more than ten years before, but seems to be missed by many investigators: there is a difference when studying collateral function in non-occluded vessels during a balloon occlusion as compared to the collateral function permanently recruited in totally occluded arteries. The latter of course requires assessment before re-establishing antegrade flow. 

The acutely recruited collaterals hardly ever achieve the same functional capacity as the collaterals permanently in function. In the paper by Pereira and colleagues the difference in collateral pressure index was 19% of the aortic pressure in non-occluded arteries, as compared to about 32% in totally occluded arteries [39]. The subsequent reocclusion in the former group may yield a similar functional capacity even within one day after treating the non-occluded lesion, whereas in the latter group of totally occluded arteries, the functional loss becomes immediately evident within 5 minutes after opening the artery. That has been shown for the first time using Doppler wires in a sequential assessment before, immediately after and one day following the recanalization of a chronically occluded artery [40].

To further elucidate the changes brought along with opening a chronically occluded artery, and the effect on both collateral function and microcirculation, we have used a combined assessment with pressure and Doppler wires [41]. There appear to be differences in the recruitability of collaterals early after angioplasty between diabetic and non-diabetic patients, the former showing a less pronounced recruitment especially when the duration of the previous occlusion was less than 3 months [42]. This could be a possible piece in the puzzle that explains the disadvantage of diabetic patients with coronary artery disease, and the higher incidence of major adverse events even with interventional therapy.

We further enhanced the observation on collateral regression after successful recanalization of a chronic occlusion by the systematic assessment of collateral function during a long-term follow-up [43]. During revaluation using a balloon reocclusion within a previously implanted stent, a further functional regression can be shown. However, the complete recovery of collateral function observed in those patients who presented with reocclusion (at the time of the study only bare metal stents were available and the reocclusion rate was considerable) suggests that collaterals remain recruitable during recurrence of an occlusion and do not disappear completely after recanalization.

It is evident that we could not perform a long-lasting reocclusion in man, and within the planned 3 minute duration, the balloon occlusion had to be relieved prematurely in some patients because of angina. Therefore the time interval required for full functional recovery in man is difficult to determine, but it appears to be longer than in the animal model. In the case of an acute reocclusion, collateral recovery would take too long to prevent ischemia in most patients as only 18% of patients had a pressure index above 0.3 at follow-up, indicating a threshold that could prevent severe ischemia during reocclusion [44].

The mechanisms involved in collateral regression and recovery are most likely flow-dependent changes of the collateral vascular tone. At baseline the microvascular arterioles and collaterals would be maximally dilated, and both collateral and peripheral conductance were high. After recanalization the peripheral arteriolar tone increases as a consequence of the autoregulatory potential of the microvasculature to respond to the improved perfusion, and conductance decreases. The much steeper decrease in collateral conductance indicates that they may collapse when they are not required after the removal of the pressure gradient between donor and recipient artery. A longer lasting reocclusion would again increase the pressure gradient and lead to a reopening of the collateral connections.

It is most notable that the fraction of patients retaining a well recruitable collateral function of 18% was close to the fraction of natural collateral connections observed in healthy arteries, indicating the presence and maintenance of preformed large interarterial connections that would provide instantaneous collateral supply [10]. No clinical determinant (presence of diabetes, prior MI) of collateral regression could be detected. There was even no correlation of the degree of restenosis with the recruitable collateral function. The immediate loss of collateral function is related to the size of the collateral connections [43]. Also after several months, the larger collaterals of connection grade 2 remain those with the greatest potential for functional recovery. 

## MULTIPLE COEXISTING COLLATERAL PATHWAYS

It should be noted, that the majority of patients with a chronically occluded coronary artery will have multiple collateral pathways, not only parallel connections for example via the septum, but also separate coexisting epicardial connections [11, 45, 46]. An occluded left anterior descending artery may receive blood supply both from the septal branches of the right posterior descending artery, as well as from epicardial branches of right ventricular branches, and as well distal connections via the posterolateral branches of the left circumflex artery. This becomes clinically relevant in case of progression of coronary artery disease or even acute occlusion during an acute myocardial infarction in one of the donor arteries [47, 48].

**Fig. (3):**
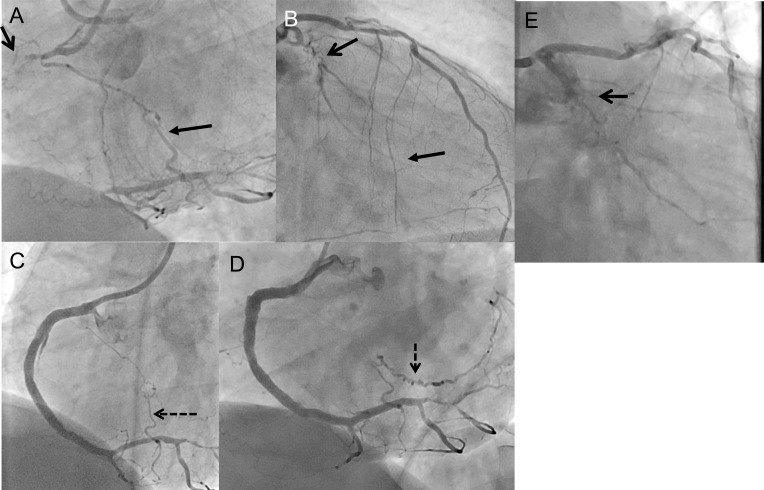
Dynamics of the angiographic appearance of collaterals. A 50 year old patient with chronic occlusion of the right coronary artery (RCA) (A, open arrow) and the circumflex artery (LCX) (B, open arrow). Collaterals supply both CTOs. The RCA CTO extends proximal until the distal bifurcation at the crux cordis, and receives both ipsilateral collaterals from the proximal RCA (A, closed arrow, CC2 size) and contralateral collaterals from the left coronary system via septal branches (B, closed arrow, CC2 size). The LCX CTO is filled distally via ipsilateral bridging collaterals along the CTO (B). The RCA was then recanalized, and the final image shows already no longer a filling of the ipsilateral collateral (C). Distally a branch is marked by a dashed arrow. Four months later the patient was re-examined to perform the recanalization of the LCX CTO. At that time the RCA branch is now fully developed as a major contralateral collateral towards the LCX CTO (D, dashed arrow), while the ipsilateral collaterals visible on B as bridging collaterals are no longer visible (E), and the antegrade filling of the LCX distal to the CTO is obscured now by contralateral collateral washout.

An example may illustrate the coexisting pathways with changing prevalence during the course of a sequential coronary revascularization (Fig. **3**). This is also clinical evidence for the observation that only the largest 2-3 collaterals provide the major collateral supply to an occluded territory based on Ohm’s law of parallel resistances [46]. The fact that several parallel collateral pathways may be “dormant” and angiographically not visible explains the observation during advanced recanalization procedures, when collateral passage with dedicated guide wires is attempted and sometimes successful through channels that were not visible initially. These channels may be constricted, but not obstructed, and therefore can be passed by hydrophilic wires [49, 50]. 

## SUMMARY

The knowledge of collateral physiology in man has increased considerably over the past two decades with the advent of intracoronary sensors of coronary pressure and flow velocity. A number of experimental studies addressed many aspects of human coronary physiology, many of these are pertinent to the everyday clinical practice in interventional cardiology. However, the human patient is not a well-defined experimental model with comorbidities and individual variations, which limits exact quantitative studies and brings along some degree of uncertainty. Nevertheless, in the absence of ideal animal models that mimic the human collateral circulation, we need to rely on studies in man.

A number of basic physiologic questions have been answered by these studies. We know that collaterals have the capacity to prevent myocardial necrosis and may even uphold metabolic supply to the territory distal to an occlusion to maintain full contractile capacity, but during exercise these collaterals are limited in their functional reserve, and more than 90% of patients with a well collateralized occlusion will experience ischemia. 

The collateral development is, in general, a process of weeks to a couple of months, however, the individual predisposition to have already easily recruitable interarterial connections determines the individual response to an occlusion, from severe and extensive myocardial infarction to a silent occlusion with completely preserved left ventricular function. Only well-developed collaterals will preserve viability, but on the other hand, collateral development does not depend on the presence of viability. So when we observe an occluded artery with excellent angiographic filling but akinesia in the supplied myocardial territory, we should base our indication for revascularization not on the quality of the collaterals but on tests of myocardial viability.

Collaterals will regress once the native artery that was replaced by the collaterals is revascularized. This process starts immediately after the re-established antegrade flow with immediate loss of collateral conductance, and extends further many months after the angioplasty or revascularization procedure. Acute reocclusion for example in the course of a late stent thrombosis would therefore lead to an acute coronary syndrome in most cases, as the recruitment of collaterals is not instantaneous.

## Figures and Tables

**Table 1.** Angiographic Definitions of Collaterals Supplying Occluded Coronary Arteries.

Rentrop classification12	Developed for occluded and non-occluded arteries
0	no filling of collateral vessels
1	filling of collateral vessels without any epicardial filling of the target artery
2	partial epicardial filling by collateral vessels of the target artery
3	complete epicardial filling by collateral vessels of the target artery
**Collateral connection grade11 **	**In CTOs, Rentrop 3 is prevalent in 85% of lesions, CC grading provides an additional differentiation**
CC0	no continuous connection
CC1	threadlike continuous connection
CC2	side branch–like connection (≥0.4mm)
CC3	>1mm diameter of direct connection (not included in the original description)
